# Thyroid Status and Sperm Abnormality Phenotypes Among Infertile Males Attending a Tertiary Referral Center in Bangladesh: A Cross-Sectional Study

**DOI:** 10.7759/cureus.115071

**Published:** 2026-08-24

**Authors:** Gufrana Afrin, Neelufar S Chowdhury, Sangeeta Mukherjee, Amit Chatterjee, Sabrina Amin, Rabiul Awal, Farzana Afrin, Sarah Ahmed

**Affiliations:** 1 Department of Biochemistry, National Institute of Laboratory Medicine and Referral Centre, Dhaka, BGD; 2 Department of Biochemistry, Dhaka Medical College, Dhaka, BGD; 3 Department of Biochemistry, Patuakhali Medical College, Patuakhali, BGD; 4 Department of Chest Diseases, Bhola Chest Disease Clinic, Bhola, BGD; 5 Department of Biochemistry, Army Medical College, Cumilla, BGD; 6 Department of Pediatric Surgery, City Medical College and Hospital, Gazipur, BGD; 7 Department of Psychiatry, National Institute of Mental Health and Hospital, Dhaka, BGD; 8 Department of Community Medicine, Medical College For Women and Hospital, Dhaka, BGD

**Keywords:** bangladesh, hypothyroidism, male infertility, semen parameters, sperm abnormality phenotypes, thyroid status

## Abstract

Background

Thyroid hormones triiodothyronine (T3) and thyroxine (T4) are recognized modulators of male reproductive physiology, acting on Sertoli cells, Leydig cells, and germ cells to regulate spermatogenesis. Despite growing evidence linking thyroid dysfunction to altered semen quality, the association between thyroid status and specific sperm abnormality phenotypes remains insufficiently characterized, with no prior data available from Bangladesh. This study aimed to evaluate thyroid status and its association with sperm abnormality phenotypes among infertile males attending a tertiary referral center in Bangladesh.

Methods

A hospital-based cross-sectional observational study was conducted at the Reproductive Endocrinology and Infertility outpatient department and In Vitro Fertilization (IVF) Center, Dhaka Medical College Hospital, Bangladesh, from January 2025 to June 2025. Ninety infertile males aged 21-50 years with confirmed abnormal semen parameters were enrolled using purposive sampling. Participants were classified into four phenotypic groups per WHO 2021 sixth edition criteria: oligospermia, isolated asthenozoospermia, oligo-asthenozoospermia, and astheno-teratozoospermia. Individuals with chronic systemic diseases independently associated with altered thyroid or reproductive function were excluded. Serum T3, T4, TSH, and follicle-stimulating hormone (FSH) were measured by chemiluminescence immunoassay (CLIA). Statistical analysis included the Kruskal-Wallis test, Mann-Whitney U test, chi-squared test, and Spearman rank correlation, using SPSS version 26 (IBM Inc., Armonk, New York).

Results

The median age was 35.0 years (IQR: 30.0-38.5). Oligospermia was the most prevalent phenotype (62.2%), followed by oligo-asthenozoospermia (22.2%) and isolated asthenozoospermia (12.2%). Hypothyroidism was disproportionately prevalent among men with oligo-asthenozoospermia (70.0%), while hyperthyroidism was more frequent among asthenozoospermic men (27.3%). Statistically significant differences in serum T3 (p=0.017), T4 (p=0.041), and TSH (p=0.003) were observed across phenotypic groups. Thyroid status was significantly associated with semen abnormality type (p<0.001). Serum T3 and T4 correlated positively with total sperm count (rs=0.427 and 0.422, respectively) and sperm morphology (rs=0.532 and 0.370), while TSH showed significant inverse correlations with both parameters. No significant correlation was observed with sperm motility.

Conclusions

Thyroid dysfunction, particularly hypothyroidism, was significantly associated with specific sperm abnormality phenotypes among infertile males in Bangladesh. Thyroid hormone levels correlated meaningfully with sperm count and morphology, suggesting that thyroid evaluation may be considered as part of the male infertility workup. Larger, multicenter, longitudinal studies are needed to confirm these associations and guide clinical practice.

## Introduction

Infertility, characterized by the inability to conceive despite twelve months of regular unprotected intercourse, represents a major reproductive and public health challenge worldwide. Worldwide, an estimated 8-12% of reproductive-aged couples are affected, and a male contribution is implicated as a sole or partial factor in roughly 50% of these cases [1]. A Global Burden of Disease 2021 analysis across 204 countries documented that the global prevalence of male infertility rose by approximately 16.9% between 1990 and 2021, with South Asia recording a substantially steeper increase of 47.2% over the same period [2]. Beyond its clinical dimensions, male infertility imposes a substantial psychosocial toll, including heightened anxiety and diminished self-esteem, which highlights the need for thorough male fertility assessment [1].

Semen analysis continues to serve as the foundation of the diagnostic evaluation for male infertility. According to the sixth edition of the WHO laboratory manual (2021), oligospermia is defined by a sperm concentration under 16 × 10⁶/mL or a total count under 39 × 10⁶ per ejaculate, asthenozoospermia by progressive motility under 32%, and teratozoospermia by normal morphology under 4% [3]. These phenotypes frequently co-exist, and their combined forms represent clinically challenging presentations. Application of the 2021 WHO criteria to a cohort of 788 infertile men demonstrated that 42.2% previously classified as normal under 2010 criteria were re-categorized as having semen abnormalities, confirming the clinical significance of updated thresholds [3].

The hypothalamic-pituitary-gonadal (HPG) axis governs spermatogenesis: GnRH triggers pituitary secretion of follicle-stimulating hormone (FSH) and luteinizing hormone (LH), which in turn act on Sertoli and Leydig cells, respectively [4]. FSH supports germ cell maturation and blood-testis barrier integrity, while LH drives Leydig cell testosterone synthesis, indispensable for spermatogenesis [4]. Disruption at any level of this axis results in defective spermatogenesis, making hormonal assessment an essential component of the infertility evaluation [1].

Thyroid hormones triiodothyronine (T3) and thyroxine (T4) are recognized modulators of male reproductive physiology. These hormones act through receptors present on Sertoli, Leydig, and germ cells, regulating spermatogenesis via androgen receptor and cAMP/PKA signaling pathways [5]. Thyroid hormones also influence sex hormone-binding globulin (SHBG) and testosterone bioavailability; elevated T4 in hyperthyroidism significantly raises SHBG, reducing free testosterone [6]. In vitro studies further demonstrated that physiological concentrations of levothyroxine significantly improved sperm mitochondrial membrane potential, progressive motility, and reduced sperm necrosis and lipid peroxidation in men with idiopathic infertility, confirming direct thyroid hormone action on sperm function [7].

Distinct effects on semen parameters have been documented for hypothyroidism and hyperthyroidism. A meta-analysis found that subclinical hypothyroidism significantly reduced progressive sperm motility, total sperm count, and morphology [8]. A cross-sectional study further demonstrated that subclinical hypothyroidism was associated with markedly lower sperm count, motility, and morphology [6]. Regarding hyperthyroidism, a prospective controlled study found significantly lower sperm motility in hyperthyroid men compared to controls, with improvement following restoration of euthyroidism [9]. A narrative review concluded that both thyroid states affect semen parameters but that published human data remain conflicting and methodologically heterogeneous [10].

Despite this evidence, the relationship between thyroid status and specific sperm abnormality phenotypes remains insufficiently defined. Many prior studies evaluated thyroid function or semen parameters in isolation, used heterogeneous populations without phenotypic stratification, or lacked adequate statistical power. In Bangladesh, infertile couples increasingly attend tertiary referral centers, with male factors identified as the obvious cause in approximately 43.3% of presenting couples [11]. While local evidence documents hypothyroidism in 23% of infertile women at tertiary centers [12], comparable data on thyroid status among infertile males with defined sperm abnormality phenotypes are absent from the Bangladeshi literature. Accordingly, the present cross-sectional study was undertaken in infertile men with abnormal semen parameters presenting to a tertiary referral center in Bangladesh. This study aimed to evaluate thyroid status and its association with sperm abnormality phenotypes among infertile males attending a tertiary referral center in Bangladesh.

## Materials and methods

Study design and setting

This hospital-based, cross-sectional observational study was carried out in the Department of Biochemistry, Dhaka Medical College (DMC), Dhaka, Bangladesh, together with the Reproductive Endocrinology and Infertility outpatient department and In Vitro Fertilization (IVF) Center under the Department of Gynecology and Obstetrics at Dhaka Medical College Hospital (DMCH). Recruitment spanned a six-month period between January 2025 and June 2025. DMCH is a major tertiary care referral center in Bangladesh providing specialized reproductive endocrinology and infertility services, including semen analysis and hormonal evaluation.

Study population

The study enrolled adult infertile men presenting with abnormal semen parameters at the Reproductive Endocrinology and Infertility OPD and IVF Center during the study period. In line with WHO criteria, infertility was taken as the inability to attain a clinical pregnancy following at least twelve months of regular, unprotected sexual intercourse [13]. Following the WHO sixth edition laboratory manual (2021), semen abnormalities were categorized as oligospermia (total sperm count <39 × 10⁶ per ejaculate), asthenozoospermia (progressive motility <32%), teratozoospermia (normal morphology <4%), or their combined forms [14]. Participants were classified into four study groups: oligospermia only (group A), isolated asthenozoospermia (group B), combined oligospermia with asthenozoospermia (group C), and astheno-teratozoospermia (group D). Men aged 21-50 years with confirmed abnormal semen parameters were eligible for inclusion. Exclusion criteria comprised secondary infertility of any identifiable cause and any history of chronic debilitating illness - malignancy, chronic kidney disease, chronic liver disease, diabetes mellitus, or hypertension - since these disorders independently affect thyroid function and semen quality and would confound interpretation of the results.

Sampling procedure and sample size

Participants meeting the predefined inclusion and exclusion criteria were recruited through a purposive sampling technique. The required sample size was derived from the standard single-population-proportion formula,

\[ n = \frac{Z^{2}pq}{d^{2}} \]

where z = 1.96 (95% confidence level), p = 0.363 corresponding to the pooled prevalence of asthenozoospermia (29.2%) and teratozoospermia (7.1%) reported among Bangladeshi infertile males by Rahman et al. [15]; q = 1 − p = 0.637; and d = 0.10 (10% margin of error). This computation yielded a minimum of 89, which was rounded up to 90 participants.

Data collection procedures

Information was gathered using a structured, pre-formed questionnaire. Following written informed consent, detailed clinical history, height, weight, smoking history, and duration of infertility were recorded. After 48-72 hours of sexual abstinence, semen was obtained by masturbation in a private hospital room or at home when travel time was within one hour, and specimens were transported at body temperature. Semen analysis was performed in the laboratory of the Department of Biochemistry, DMC, applying the 2021 WHO sixth edition reference parameters [14].

For thyroid evaluation, five milliliters of venous blood were drawn under aseptic conditions, left to clot for 30 minutes, and then centrifuged at 3000 rpm for 10 minutes. The separated serum was aliquoted and kept at -20°C until analysis. Serum triiodothyronine (T3), thyroxine (T4), thyroid-stimulating hormone (TSH), and follicle-stimulating hormone (FSH) were quantified by the Chemiluminescence Immunoassay (CLIA) method. The applied reference intervals were 3.1-6.8 pmol/L for serum T3, 12-22 pmol/L for serum T4, and 0.27-4.20 mIU/L for serum TSH [16]. In accordance with American Thyroid Association reference criteria, thyroid status was classified as euthyroid (TSH 0.27-4.20 mIU/L), hypothyroid (TSH >4.20 mIU/L), or hyperthyroid (TSH <0.27 mIU/L) [17]. All hormone assays (serum T3, T4, TSH, and FSH) were performed on a single automated chemiluminescence immunoassay analyzer (MAGLUMI 800, Snibe Diagnostics, Shenzhen, China) using the manufacturer's dedicated reagent kits, calibrators, and controls, strictly according to the manufacturer's instructions. Manufacturer-supplied internal quality-control materials at two concentration levels (normal and abnormal) were analyzed at the start of each working run, and patient results were accepted only when control values fell within the manufacturer's defined ranges. The intra-assay and inter-assay coefficients of variation were maintained below 5% and 8%, respectively, for all analytes. All samples were processed in a single laboratory (Department of Biochemistry, Dhaka Medical College) by the same trained personnel to minimize inter-operator and inter-run variability. Thyroid status was defined primarily on the basis of serum TSH, while serum T3 and T4 were used to further characterize the biochemical thyroid profile; overt and subclinical thyroid dysfunction were not separately sub-classified, and thyroid autoantibodies were not measured.

Statistical analysis

Data were verified for completeness and processed in the Statistical Package for the Social Sciences (SPSS), version 26 (IBM Inc., Armonk, New York). Non-normally distributed continuous variables were reported as median with interquartile range (IQR), whereas categorical variables were presented as frequencies and percentages. The Shapiro-Wilk test was applied to assess normality. Between-group comparisons were performed using the Kruskal-Wallis test for overall differences across the four phenotype groups. Associations among categorical variables were tested with the chi-squared test. Spearman's rank correlation coefficient (rₛ) was used to evaluate relationships between thyroid hormone levels and clinical and semen parameters. Statistical significance was set at a p-value below 0.05. Potential confounding was addressed principally at the design stage through the predefined exclusion of chronic systemic illnesses known to influence thyroid or reproductive function. Given the limited overall sample size and the small number of participants within individual phenotype subgroups, multivariable-adjusted modeling was not undertaken; the associations reported here are therefore unadjusted.

Ethical considerations

The study protocol received ethical clearance from the Institutional Review Board (IRB) of Dhaka Medical College, Dhaka (approval no: IRB-DMC/2024/391). All procedures adhered to the principles of the Declaration of Helsinki. Every participant provided written informed consent before enrollment. Participant data were kept strictly confidential and anonymous throughout the study, and all participants were informed of their right to withdraw at any stage without consequences.

## Results

Participants had a median age of 35.0 years (IQR: 30.0-38.5); the 35-39 years age group was the largest at 25 (27.8%), followed by 30-34 years, 22 (24.4%), 25-29 years, 21 (23.3%), 40-44 years, 18 (20.0%), and ≥45 years, four (4.4%). No participants in this cohort were aged 21-24 years, although this age range fell within the study’s inclusion criteria. Regarding body mass index, the median BMI was 24.2 kg/m² (IQR: 22.0-24.2) among those with normal weight, 17.8 kg/m² (IQR: 17.3-18.4) among underweight participants, and 26.0 kg/m² (IQR: 25.6-27.0) among overweight participants. The majority of participants were non-smokers, 51 (56.7%), while 39 (43.3%) were smokers. In terms of duration of infertility, more than half had experienced infertility for 5-10 years, 55 (61.1%), followed by 11-15 years, 21 (23.3%), and less than five years, 14 (15.6%) (Table 1).

**Table 1:** Demographic characteristics of study participants (n=90) Data presented as frequency and percentage; BMI presented as median (IQR)

Variable	Category	n (%)
Age (years)	25-29	21 (23.3)
	30-34	22 (24.4)
	35-39	25 (27.8)
	40-44	18 (20.0)
	≥45	4 (4.4)
Median age (IQR) years	35.0 (30.0-38.5)	
BMI (kg/m²), median (IQR)	Underweight	17.8 (17.3-18.4)
	Normal	24.2 (22.0-24.2)
	Overweight	26.0 (25.6-27.0)
Smoking status	Smoker	39 (43.3)
	Non-smoker	51 (56.7)
Duration of infertility	<5 years	14 (15.6)
	5-10 years	55 (61.1)
	11-15 years	21 (23.3)

The median sperm motility among participants was 40.0% (IQR: 30.0-83.7%), and the median total sperm count was 15.0 million (IQR: 8.5-28.0 million). Sperm morphology had a median value of 30.0% (IQR: 4.0-40.0%). Regarding thyroid hormones, the median serum T3 was 1.70 nmol/L (IQR: 0.86-2.38), median serum T4 was 66.13 nmol/L (IQR: 56.65-78.74), and median serum TSH was 1.37 mIU/L (IQR: 0.73-5.77). The median serum FSH level was 1.77 IU/L (IQR: 1.20-3.58) (Table 2).

**Table 2:** Semen parameters and serum hormone levels among study participants (n=90) Data presented as Median (IQR). T3 - triiodothyronine; T4 - thyroxine; TSH - thyroid-stimulating hormone; FSH - follicle-stimulating hormone

Parameter	Median	IQR
Sperm motility (%)	40.0	30.0-83.7
Total sperm count (million)	15.0	8.5-28.0
Sperm morphology (%)	30.0	4.0-40.0
Serum T3 (nmol/L)	1.70	0.86-2.38
Serum T4 (nmol/L)	66.13	56.65-78.74
Serum TSH (mIU/L)	1.37	0.73-5.77
Serum FSH (IU/L)	1.77	1.20-3.58

Among the study participants, oligospermia was the most prevalent sperm abnormality, affecting 56 (62.2%) of the subjects. Oligospermia with asthenozoospermia was the second most common finding, observed in 20 (22.2%) of participants. Isolated asthenozoospermia was identified in 11 (12.2%), while astheno-teratozoospermia was the least common, occurring in only three (3.3%) of participants (Table 3).

**Table 3:** Distribution of sperm abnormalities among study participants (n=90) Data presented as frequency (n) and percentage (%).

Type of sperm abnormality	n	%
Oligospermia	56	62.2
Oligospermia with asthenozoospermia	20	22.2
Asthenozoospermia	11	12.2
Astheno-teratozoospermia	3	3.3

Table 4 presents how serum thyroid hormone concentrations relate to the different types of sperm abnormalities. Statistically significant differences were observed across all three thyroid hormones. Serum T3 varied significantly among the sperm abnormality groups (p=0.017); the highest median T3 was recorded in the asthenozoospermia group (2.38 nmol/L; IQR: 2.08-2.80) and the lowest in the oligo-asthenozoospermia group (0.86 nmol/L; IQR: 0.72-2.87). A significant group difference was likewise seen for serum T4 (p=0.041), with the asthenozoospermia group again exhibiting the highest median T4 (74.47 nmol/L; IQR: 67.40-152.71). Serum TSH demonstrated the most significant variation across groups (p=0.003), with the oligo-asthenozoospermia group showing the highest median TSH (5.78 mIU/L; IQR: 0.39-5.90) and the asthenozoospermia group the lowest (0.50 mIU/L; IQR: 0.38-0.78).

**Table 4:** Association of serum thyroid hormone levels with types of sperm abnormalities Data presented as median (IQR). *Statistically significant (p < 0.05). Kruskal–Wallis test used for overall comparison across groups.

Sperm Abnormality	T3 median		T4 median		TSH median	
	(nmol/L)	IQR	(nmol/L)	IQR	(mIU/L)	IQR
Oligospermia (n=56)	1.7	0.87-2.33	66.13	56.08-78.74	1.37	0.76-5.70
Asthenozoospermia (n=11)	2.38	2.08-2.80	74.47	67.40-152.71	0.5	0.38-0.78
Oligo + asthenozoospermia (n=20)	0.86	0.72-2.87	58.32	55.09-151.61	5.78	0.39-5.90
Astheno-teratozoospermia (n=3)	1.23	1.23-1.23	68.84	68.80-68.84	2.63	2.63-2.63
p-value (Kruskal-Wallis)	0.017*		0.041*		0.003*	

The distribution of thyroid status across the semen abnormality types showed a strongly significant association (p<0.001) (Table 5). Among participants with oligospermia, the majority were euthyroid 29 (51.8%), followed by hypothyroid 21 (37.5%) and hyperthyroid, six (10.7%). In contrast, all participants with astheno-teratozoospermia were euthyroid, three (100.0%). Among those with asthenozoospermia, most were euthyroid, eight (72.7%), with the remaining three (27.3%) being hyperthyroid and none being hypothyroid. Notably, hypothyroidism was predominantly associated with the oligo-asthenozoospermia group, where 14 (70.0%) of participants were hypothyroid, compared to none in the asthenozoospermia group.

**Table 5:** Association between thyroid status and semen abnormalities Data presented as n (%). *Statistically significant (p < 0.001). Fisher's exact test was used.

Thyroid status	Oligospermia (n=56)	Asthenozoospermia (n=11)	Oligo + asthenozoospermia (n=20)	Astheno-teratozoospermia (n=3)
Euthyroid	29 (51.8%)	8 (72.7%)	0 (0.0%)	3 (100.0%)
Hypothyroid	21 (37.5%)	0 (0.0%)	14 (70.0%)	0 (0.0%)
Hyperthyroid	6 (10.7%)	3 (27.3%)	6 (30.0%)	0 (0.0%)
p-value	< 0.001*			

Spearman correlation analysis revealed several significant associations between thyroid hormones and clinical and semen parameters (Table 6). BMI correlated strongly and significantly with all three thyroid hormones - inversely with T3 (rₛ=-0.699, p<0.001) and T4 (rₛ=-0.713, p<0.001), and positively with TSH (rₛ=0.758, p<0.001). Serum FSH showed positive correlations with T3 (rₛ=0.411, p=0.002) and T4 (rₛ=0.294, p=0.028) but no significant relationship with TSH. Total sperm count rose significantly with T3 (rₛ=0.427, p<0.001) and T4 (rₛ=0.422, p<0.001) and fell significantly with TSH (rₛ=-0.506, p<0.001). A comparable pattern emerged for sperm morphology, which was positively linked to T3 (rₛ=0.532, p<0.001) and T4 (rₛ=0.370, p<0.001) and negatively linked to TSH (rₛ=-0.423, p<0.001). In contrast, age, duration of infertility, and sperm motility bore no statistically significant correlation with any of the thyroid hormone levels.

**Table 6:** Spearman correlation of serum thyroid hormone levels with clinical and semen parameters rs - Spearman correlation coefficient. *p < 0.05; **p < 0.001

Variable	Serum T3		Serum T4		Serum TSH	
	rs	p	rs	p	rs	p
Age (years)	0.108	0.429	0.227	0.092	-0.169	0.212
BMI (kg/m²)	-0.699	<0.001**	-0.713	<0.001**	0.758	<0.001**
Duration of infertility	0.029	0.789	0.094	0.379	-0.063	0.556
Serum FSH (IU/L)	0.411	0.002**	0.294	0.028*	-0.133	0.328
Total sperm count (million)	0.427	<0.001**	0.422	<0.001**	-0.506	<0.001**
Sperm morphology (%)	0.532	<0.001**	0.370	<0.001**	-0.423	<0.001**
Sperm motility (%)	-0.143	0.244	−0.058	0.641	0.085	0.493

## Discussion

This study examined the relationship between thyroid status and sperm abnormality phenotypes among infertile men attending a tertiary referral center in Bangladesh. The present study demonstrated that oligospermia was the most common sperm abnormality phenotype, followed by combined oligo-asthenozoospermia, isolated asthenozoospermia, and astheno-teratozoospermia. Euthyroidism was the most frequently observed overall thyroid status, although hypothyroidism represented the most prevalent thyroid dysfunction. A significant association was observed between thyroid status and specific sperm abnormality phenotypes, with hypothyroidism disproportionately represented among men with oligospermia and oligo-asthenozoospermia. Serum T3, T4, and TSH correlated significantly with total sperm count and sperm morphology, whereas no significant correlation emerged with sperm motility.

The predominance of oligospermia in this cohort is consistent with previous reports from infertility clinics in South Asia and other low- and middle-income settings. Tertiary-center data from Bangladesh similarly identified oligozoospermia and severe oligospermia as leading semen abnormalities among men presenting for infertility evaluation [18], while data from South India and Nigeria also reported oligozoospermia and its combined forms as substantial contributors to abnormal semen findings [19,20]. Plausible explanations include impaired spermatogenesis secondary to endocrine dysfunction, environmental and occupational exposures, oxidative stress affecting germ cell integrity, smoking, and delayed presentation to fertility services, all of which may compound subclinical reproductive impairment before diagnosis.

A relatively high burden of thyroid dysfunction was noted in this infertile male population, with hypothyroidism emerging as the most common disorder. This observation is partially concordant with earlier studies from Pakistan and Nigeria that reported thyroid hormone abnormalities among infertile men with altered sperm parameters [6,20]. Thyroid hormones are biologically important regulators of testicular development, acting on Sertoli cell maturation and proliferation through thyroid hormone receptors and modulating Leydig cell steroidogenesis and testosterone production [21,22]. Through crosstalk linking the hypothalamic-pituitary-thyroid and hypothalamic-pituitary-gonadal axes, these effects point to a plausible contribution of thyroid hormones to spermatogenesis [23,24].

The principal finding of this study was the significant link between thyroid status and particular sperm abnormality phenotypes. Oligospermic and oligo-asthenozoospermic men were more frequently hypothyroid, whereas asthenozoospermic men showed comparatively lower TSH and higher T3/T4 levels, a pattern that may reflect phenotype-specific endocrine mechanisms. This is biologically plausible, as hypothyroidism may adversely affect spermatogenesis through impaired morphology and motility [25], whereas hyperthyroid states may influence sperm count and motility through altered sex hormone-binding globulin and androgen bioavailability [9]. However, the available evidence remains heterogeneous; an Italian andrology-clinic study reported no meaningful relationship between thyroid hormone levels and conventional semen parameters [26]. This discrepancy may reflect differences in study population, sample size, phenotype classification, and prevalence of overt thyroid dysfunction. Potential mechanisms include thyroid hormone-mediated regulation of germ cell differentiation, epididymal maturation, and oxidative stress pathways within the testicular microenvironment [23,27]. These phenotype-specific associations should nonetheless be interpreted with caution, given the modest overall sample size, the very small number of participants in some subgroups (notably astheno-teratozoospermia, n=3), the unadjusted nature of the analyses, and the possibility of residual confounding from unmeasured factors.

The positive correlations between T3/T4 and both total sperm count and sperm morphology, together with the inverse correlation for TSH, are consistent with previous reports linking thyroid status to semen quality. A meta-analytic synthesis reported measurable reproductive alterations in subclinical hyperthyroidism [28], while clinical studies of hypothyroid men showed that sperm morphology was particularly affected and improved after levothyroxine treatment [25]. These associations may be explained by thyroid hormone effects on Sertoli cell metabolic support, Leydig cell-mediated testosterone synthesis, mitochondrial function, and sperm maturation [22,25]. However, the absence of significant correlation with sperm motility suggests that this relationship may be parameter-specific, with motility also influenced by epididymal function, infection, inflammation, mitochondrial integrity, and oxidative stress.

The inverse relationship between BMI and T3/T4, together with the positive association between BMI and TSH, is consistent with studies describing obesity-related alterations in thyroid function [29,30]. The positive correlations between FSH and both T3 and T4 may reflect interaction between thyroid and gonadal endocrine axes.

To our knowledge, this is among the first studies from Bangladesh to investigate the relationship between thyroid status and specific sperm abnormality phenotypes in infertile males, using phenotypic classification grounded in the WHO 2021 sixth edition semen analysis criteria. Hormonal assessment included all three thyroid parameters (T3, T4, and TSH) alongside FSH, enabling a comprehensive endocrine profile. However, several limitations apply. Its cross-sectional, single-center design and relatively small sample size (n=90) restrict generalizability and preclude causal inference. Data relied on purposive sampling, introducing selection bias. The astheno-teratozoospermia group was markedly small (n=3), which may reduce the stability of between-group comparisons involving this subgroup. Semen analysis was performed on a single sample, which may not reflect intraindividual biological variability. Unmeasured confounders including nutritional status, oxidative stress markers, environmental and occupational exposures, and prior reproductive history may represent residual confounding. Longitudinal data on thyroid or semen parameter trajectories over time were not assessed, limiting mechanistic interpretation of observed associations. In addition, thyroid status was characterized using standard biochemical thresholds without separating subclinical from overt dysfunction or measuring thyroid autoantibodies, which limited detailed phenotyping of thyroid disorders. Full in-house laboratory quality-control metrics, such as measured assay coefficients of variation, were not independently established beyond manufacturer specifications. No multivariable adjustment was performed, so the reported associations are unadjusted and may be subject to residual confounding. Finally, the absence of a healthy or fertile control group precludes comparison with a normozoospermic reference population and limits interpretation of the observed thyroid-semen associations.

## Conclusions

In this study, thyroid dysfunction, particularly hypothyroidism, was significantly associated with specific sperm abnormality phenotypes among infertile males, with oligo-asthenozoospermia showing the strongest association. Serum T3 and T4 were positively related to total sperm count and morphology, whereas TSH correlated inversely, suggesting that thyroid hormones may influence spermatogenesis. However, as these analyses were unadjusted and based on a modest sample with small subgroups, the findings should be regarded as hypothesis-generating rather than confirmatory. These results nonetheless imply that thyroid assessment could be considered as part of the male infertility work-up, pending confirmation in larger studies. Larger multicenter, longitudinal investigations that incorporate a broader range of reproductive and endocrine parameters are needed to confirm these associations and to guide targeted therapeutic strategies in this population.
